# Outcomes of Succinylcholine and Rocuronium for Rapid Sequence Intubation in the Emergency Department

**DOI:** 10.5811/westjem.50495

**Published:** 2026-05-03

**Authors:** Danielle H. O’Connell, Joseph Yeager, Rebecca A. Abrams, John R. Zatarain, Krishna K. Paul, Rekha R. Goswami, Kelsey Hill, Lisa R. Farmer, Julio Jayes, Dietrich VK. Jehle

**Affiliations:** * University of Texas Medical Branch, Department of Emergency Medicine, Galveston, Texas; † University of Texas Medical Branch, Department of Anesthesiology, Galveston, Texas

## Abstract

**Introduction:**

Succinylcholine and rocuronium are neuromuscular blocking agents commonly used as paralytics in the emergency department (ED) during rapid sequence intubation. Prior studies have shown mixed results regarding the preferred agent aside from settings where there are contraindications. This study compares outcomes of death, myocardial infarction, and post-traumatic stress disorder for succinylcholine vs rocuronium when used in rapid sequence intubation using data from a large, multicenter database.

**Methods:**

In this retrospective study, we extracted 105 million patient records from 61 healthcare organizations in the United States from the TriNetX database between 2004–2023. Adults ≥ 18 years of age who underwent intubation on the same day as an ED visit and received succinylcholine or rocuronium with the hypnotic anesthetic etomidate were included. The outcomes evaluated were mortality and myocardial infarction within 60 days after intubation. We excluded patients with prior history of myocardial infarction. We performed propensity matching for demographics and nine pre-existing conditions associated with mortality.

**Results:**

There were 15,514 patients in the succinylcholine group and 14,675 patients in the rocuronium group for a total of 30,189 adults prior to propensity matching. The final cohort included 26,884 patients evenly divided between groups after propensity matching. Patients given succinylcholine were associated with a significantly lower risk of mortality (30.1% vs 33.4%, risk ratio [RR] 0.901, 95% CI, 0.869–0.933, P < .001, absolute risk reduction of 3.3%) and myocardial infarction (10.5% vs 11.9%, RR 0.888, 95% CI, 0.828–0.953, P = .001, absolute risk reduction of 1.4%) within 60 days after rapid sequence intubation. Trends were similar before propensity matching.

**Conclusion:**

Succinylcholine administration was associated with reduced mortality compared to rocuronium. These findings suggest succinylcholine may be a safer paralytic agent for rapid sequence intubation when no contraindications are identified.

## INTRODUCTION

### Background

Succinylcholine and rocuronium are the most common paralytics used in the emergency department (ED) for rapid sequence intubation (RSI), and their use improves intubation conditions.^1^,^2^ Succinylcholine is a short-acting, depolarizing neuromuscular agent with standard dosing of 1.0–1.5 mg per kilogram (kg) intravenous (IV) push with onset of 30–60 seconds and duration of approximately 5–10 minutes under normal circumstances.^3^ Rocuronium is a longer acting, non-depolarizing neuromuscular agent with dosing of 0.6–1.2 mg/kg IV push with onset of 45–60 seconds and duration of 20–60 minutes.^4^

When administered, these drugs work by different mechanisms to block cholinergic receptors at the neuromuscular junction, causing paralysis. During the process, both drugs can elevate heart rate or cause allergic reactions.^5^,^6^ Succinylcholine commonly causes muscle fasciculations, jaw rigidity, elevated intracranial pressure, elevated ocular pressure, or hyperkalemia, whereas rocuronium is not associated with these side effects.^3^ The hyperkalemia that occurs with succinylcholine usually peaks 2–4 minutes after administration and returns to normal within 10 minutes. The degree of potassium increase is usually 0.5–1.0 milliequivalents per liter (mEq/L) but can be higher in high-risk patients such as those with pre-existing hyperkalemia or neuromuscular disease.^3^,^7^ In addition, succinylcholine can rarely induce malignant hyperthermia.^1^

The search to determine the best paralytic for intubation is of particular interest for critically ill patients treated in the ED. The data varies, with some studies reporting that rocuronium leads to higher mortality rates while others state there is no true difference between rocuronium and succinylcholine.^8^,^9^ In addition, some authors have suggested that there is a higher incidence of post-traumatic stress disorder (PTSD) with patients receiving longer acting paralytics and inadequate sedation.^10^,^11^

### Importance

Several studies have attempted to determine which paralytic is superior overall; however, few randomized controlled trials are available. Most existing trials were conducted in inpatient settings, generally in the operating room (OR), and their primary endpoint was the assessment of intubation conditions rather than first-attempt intubation success.^12^–^15^ In addition, none of the existing studies clearly established which paralytic is best for intubation in the emergency setting.

According to the 2022 American Society of Anesthesiologists Practice Guidelines for Management of the Difficult Airway, “the literature is currently insufficient to determine the actual benefit or harm of rocuronium versus succinylcholine for airway management of anticipated difficult airway patients.”^8^ To our knowledge, no previous study of this magnitude (both sample size and multicenter design) has compared these two drugs for RSI used in the ED for critical outcomes of death and myocardial infarction.

**Population Health Research Capsule**

What do we already know about this issue?

*Succinylcholine and rocuronium are commonly used for ED rapid sequence intubation, but prior small studies show mixed outcomes and no clear mortality superiority*.

What was the research question?

*In 26,884 propensity matched patients undergoing ED RSI, does succinylcholine vs rocuronium differ in mortality or MI?*

What was the major finding of the study? *Succinylcholine had lower mortality (RR 0.90, 95% CI 0.87–0.93; p<0.001) and MI rates (RR 0.89, 95% CI, 0.83–0.95, P = .001)*.

How does this improve population health?

*This study suggests succinylcholine may be a safer paralytic agent for rapid sequence intubation when compared with rocuronium*.

### Goal of Investigation

In this study we aimed to compare outcomes of mortality, myocardial infarction, and PTSD in patients undergoing rapid sequence intubation with succinylcholine vs rocuronium in the ED from a large, real-world database.

## METHODS

### Study Design and Setting

This retrospective, propensity-matched study used de-identified medical records from 61 large healthcare organizations within the United States Collaborative Network of TriNetX. Using *International Classification of Diseases*, *10^th^ Rev, Clinical Modification* (*ICD-10-CM*) diagnosis codes, Current Procedural Terminology (CPT) codes, and RxNorm medication codes as search criteria, the database can be queried to establish multiple cohorts and compare outcomes of interest among those cohorts. Since there was no primary chart review, limitations of retrospective chart review identified by Worster and Bledsoe did not apply.^16^

### Selection of Participants

The query, conducted on August 22, 2023, included all patients in the U.S. Collaborative Network of TriNetX from January 1, 2004–June 30, 2023. We established two cohorts of patients intubated in the ED: Cohort 1 with 15,514 patients intubated using succinylcholine; and Cohort 2 with 14,675 patients intubated with rocuronium. The following billing codes were required to meet inclusion criteria (index event): intubation, endotracheal, emergency procedure (UMLS:CPT:31500); etomidate (NLM:RXNORM:4177); and succinylcholine for Cohort 1 (NLM:RXNORM:10154), or rocuronium for Cohort 2 (NLM:RXNORM:68139). We excluded patients if the index event occurred > 20 years from the time of analysis.

### Outcomes

Once the cohorts were established, we compared outcomes within the TriNetX database. Outcomes of interest were as follows: 1) death; 2) myocardial infarction (*ICD-10-CM*:I21); and 3) post-traumatic stress disorder (*ICD-10-CM*:F43.10) that occurred in a time window of 60 days following the index event: emergent intubation. Mortality data within the TriNetX platform is obtained from electronic healthcare data and healthcare organizations, in conjunction with national death registries. Patients were excluded if outcomes of myocardial infarction or PTSD occurred prior to the rapid sequence intubation.

### Post-hoc Analysis

We ran a similar analysis using data from 2018–2025 including patients given both etomidate and ketamine as induction agents and evaluating outcomes on day 0–60 to control for recent changes in intubation practices in the ED.^17^,^18^ Additionally, we analyzed the proportion of adult patients in the succinylcholine and rocuronium cohorts, presenting to the ED with *ICD-9/10-CM* codes associated with traumatic mechanisms, poisonings, and burns at the time of RSI and given etomidate as an induction agent.

### Statistical Analysis

Using linear and logistic regression, we performed a 1:1 propensity score match for both groups—patients intubated with succinylcholine and those intubated with rocuronium, for the variables of age, sex, race/ethnicity, and several pre-existing diagnoses that are risk factors for mortality (Table 1). These pre-existing conditions were chosen based on common causes of death, according to the U.S. Centers for Disease Control and Prevention.^19^ We used greedy nearest neighbor matching with a tolerance (caliper width) of 0.1 and propensity scores with a standard difference ≤ 0.1 considered a good match. The order of data rows is randomized through TriNetX to mitigate bias introduced by the nearest-neighbor algorithm. We made comparisons between cohorts before and after propensity matching. The analysis compared the outcomes of Cohort 1 and Cohort 2. After propensity matching, none of the covariates were statistically different (Table 1).

The measure of association is a statistical tool inherent in TriNetX and was used to perform univariate analysis where risk ratios (RR), 95% confidence intervals, and probability values (*P*) were calculated to compare outcomes. To help visualize survival, we ran a Kaplan-Meier curve through the database, plotting the survival probability against the number of days following an index event. The univariate analysis used the chi-square test to evaluate dichotomous data and the *t*-test to examine continuous datasets. Statistical significance was set at a two-sided alpha < 0.05. Using data from TriNetX does not require review by the University of Texas Medical Branch Institutional Review Board (IRB) as this was secondary analysis of de-identified data. The IRB determined that this project is considered “not human subjects research.”

## RESULTS

### Characteristics of Study Subjects

Before propensity matching, the analysis found 30,189 adult patient-intubation encounters with etomidate and succinylcholine (n = 15,514) or etomidate and rocuronium (n = 14,675). After propensity matching, 13,442 patient encounters were found for each cohort. Demographics used for propensity matching included race, sex, and underlying medical conditions. The mean age of patients before and after propensity matching was 54.5 and 56.0, respectively, in the succinylcholine cohort, and 56.7 and 55.9 in the rocuronium cohort. The majority of patients in both cohorts were White (not Hispanic or Latino) and male with varying degrees of underlying medical conditions. About one-fourth of patients studied had chronic lower respiratory diseases, and around 40% carried a diagnosis of hypertension. A summary of the demographic information used for propensity matching is listed in Table 1. The standard differences for all our covariates were less that < 0.02 in the analysis, representing a very strong propensity match.

### Main Results

Within 60 days of emergency intubation, after propensity matching, the two cohorts were associated with the following risk of death: 30.1% in the succinylcholine group (3,977 of 13,213 patients) vs 33.4% in the rocuronium group (4,433 of 13,266 patients) with a significant risk difference, favoring succinylcholine (RR 0.901, 95% CI, 0.869–0.933, *P* < .001, absolute risk reduction of 3.3%). The risk of myocardial infarction for the succinylcholine cohort was 10.5% (1,303 of 12,377 patients) vs 11.9% (1,454 of 12,264 patients) in the rocuronium group (RR 0.888, 95% CI, 0.828–0.953, *P* <.001 absolute risk reduction of 1.4%) (Table 2). There was no significant difference in the associated risks of PTSD (RR 1.000, 95% CI, 0.827–1.210, *P* = 1.00) between both groups. Before propensity matching, patients intubated with succinylcholine had an associated risk of death of 29.3% vs 34.1% in the rocuronium group (RR 0.832, 95% CI, 0.832–0.889, *P* < .001). The risk of myocardial infarction for succinylcholine vs rocuronium, respectively, was 10.2% vs 11.8% (RR 0.862, 95% CI, 0.806–0.922, *P* < .001). These findings are listed in Table 2.

### Post-Hoc Results

Results of the post-hoc analysis from 2018–2025 showed similar trends as the primary analysis. After propensity matching, mortality in the succinylcholine cohort was 28.5% vs 30.3% in the rocuronium cohort (RR 0.94; 95% CI 0.91–0.97; *P* < .001). The succinylcholine cohort had 11.7% patients with myocardial infarction vs 13.1% patients in the rocuronium group (RR 0.90; 95% CI 0.84–0.95; *P* < .001) (Supplementary Table 1). In the 2018–2025 post-hoc analysis, 16,929 patients received succinylcholine and 27,537 received rocuronium. This was in contrast to our initial analysis of data from 2004–2023, where succinylcholine was used in 15,514 patients and rocuronium in14,675 patients.

When evaluating the same-day outcomes for the patients from 2004–2023, approximately 25.7% of the succinylcholine cohort and 28.5% of the rocuronium cohort presented to the ED with a traumatic mechanism at the time of RSI. Overdoses represented 14.9% of the succinylcholine cohort and 15.7% of the rocuronium cohort. There was a larger gap in the percentage of patients with burns, with 1.2% in the succinylcholine cohort and 2.3% in the rocuronium cohort. In addition, our study population had an episode of malignant hyperthermia and, of interest, it was in the rocuronium group.

## DISCUSSION

This study demonstrates an overall association between the use of succinylcholine for RSI with lower mortality and myocardial infarction rates compared to rocuronium in a large, multicenter database. Following propensity score matching, the 60-day mortality rate was 30.1% in the succinylcholine cohort vs 33.4% in the rocuronium cohort. Similarly, the incidence of myocardial infarction was 10.5% with succinylcholine compared to 11.9% with rocuronium, suggesting a potential advantage for succinylcholine in critically ill adult patients undergoing RSI.

We chose a 60-day follow-up period to ensure complete ascertainment of outcomes after RSI. While Kaplan-Meier curves showed most deaths occurred within the first five days and additional events continued through approximately 30 days, extending the window to 60 days allowed capture of late mortality among patients discharged to long-term care after an anoxic injury. Although myocardial infarctions were concentrated within the first three days, using a uniform 60-day period across all endpoints provided methodological consistency and minimized the risk of underestimating clinically important late events.

These findings are consistent with a 2017 Cochrane review, which demonstrated that succinylcholine provided superior intubating conditions compared to rocuronium when assessed using the Goldberg scale.^2^ Although higher doses of rocuronium (1.2 mg/kg) have been shown to provide intubating conditions comparable to standard-dose succinylcholine and may approach similar onset times, most studies still report a modestly faster onset with succinylcholine (1.0 mg/kg), with the difference being substantially smaller than that observed with lower dose rocuronium (0.6 mg/kg).^2^ Most authors agree that the higher dose (1.2 mg/kg) of rocuronium is the appropriate dose if used for RSI. Importantly, higher dose rocuronium is also associated with a markedly prolonged duration of paralysis, which often exceeds 60 minutes. Compared with the 5–8 minutes duration typical of succinylcholine, this may be clinically relevant in scenarios involving failed or difficult intubation. In contrast, the present findings diverge from those of a recent smaller secondary analysis that reported no significant differences in severe complications between the two agents.^20^

A major strength of this study lies in the large sample size derived from the TriNetX database, making it over six times larger than any previous single study or meta-analysis on this topic. This robust dataset offers increased power to detect clinically meaningful differences. The prior National Emergency Airway Registry (NEAR) study using a multicenter registry with 4,075 patients showed that first-pass intubation success and peri-intubation adverse events are generally comparable between succinylcholine and rocuronium when rocuronium is administered at higher doses (1.0–1.2 mg/kg). In contrast, our study included a cohort approximately 7–8 times larger and evaluated downstream clinical outcomes, specifically 60-day mortality and myocardial infarction, rather than immediate airway performance. Intubation for traumatic mechanisms are associated with higher mortality.

It should be noted that in the NEAR database, traumatic intubations were 1.5 times more common in the succinylcholine group than in the rocuronium group, suggesting that the succinylcholine group was at higher risk of poor outcomes and cardiac arrest. In this study, the percentages of trauma intubation in the succinylcholine and rocuronium group were equivalent. Therefore, our findings are not refuted by prior studies that had imbalances in the cohorts.^9^ Our findings also differ from earlier studies, such as those by Marsh et al (2011) and DeMasi et al (2025), which reported no significant mortality difference between the agents, despite imbalances in baseline comorbidities that were not controlled. These are adjusted for in our study with propensity matching. Similarly, Nguyen et al (2019) found no difference in mortality or disposition but reported more intensive care unit (ICU) and ventilator days associated with succinylcholine, favoring rocuronium in terms of secondary outcomes.^14^,^15^,^20^

It is important to acknowledge key pharmacologic differences between these agents. Succinylcholine’s rapid onset and short duration may be advantageous in emergent airway scenarios, allowing for quicker recovery of spontaneous ventilation than rocuronium. Quicker recovery of spontaneous respiration makes a prolonged hypoxic injury less likely. In contrast, rocuronium’s prolonged effect may complicate cases where re-assessment or reversal of paralysis is necessary. This could potentially increase psychological distress or the risk of awareness under paralysis. However, our analysis did not detect a significant difference in PTSD outcomes between the two cohorts. This may be due to limitations in PTSD documentation and assessment within EHRs and inherent variability in clinical practices. While the ED Awareness 1 and upcoming ED Awareness 2 trials have provided foundational data in this area, their use of structured interviews and validated questionnaires cannot be replicated within the constraints of a retrospective, EHR-based database such as TriNetX.^21^

Certain patient populations—including those with hyperkalemia, traumatic brain injury, intracranial hemorrhage, known intracranial hypertension, intraocular pressure, renal failure, malignant hyperthermia risk, Guillain-Barré syndrome, spinal cord injury, or burns > 24 hours old—represent contraindications to succinylcholine. Succinylcholine administration typically causes a small, transient rise in serum potassium in healthy patients, with increases peaking within about 2–4 minutes and generally resolving within 10 minutes; markedly higher increases can occur in patients with pre-existing hyperkalemia or aforementioned contraindications.^3^,^22^,^23^ Importantly, in patients with acute burns or spinal cord injuries, clinically significant hyperkalemia does not occur immediately but rather develops days to weeks after injury, making this risk less relevant during initial ED presentations. Rocuronium is often preferred in cases associated with poor outcomes. Notably, this study was propensity-matched for one of the most common contraindications to succinylcholine, acute and chronic renal failure. These conditions are frequently linked to hyperkalemia in the ED, minimizing differences between the groups.^15^

## LIMITATIONS

Although this study found a significant association of lower mortality for succinylcholine vs rocuronium administration for RSI, the retrospective nature prevents the identification of causation. Additionally, although we used propensity matching for certain demographics, the patient population was limited to individuals ≥ 18 years of age who were intubated on the same day as an ED visit and received etomidate for induction. Beyond the nine selected comorbid conditions, other unaccounted-for conditions or markers of critical illness (such as Injury Severity Score or Acute Physiology and Chronic Health Evaluation scoring) may exist that could introduce confounding or bias. In addition, outside of protocols, clinician preference and familiarity are often a driving force in selection of interventional medications, including paralytics given for RSI. Differences in co-administered medications may contribute to variances in outcomes, but this was not analyzed.

There is potential for missed death events when a patient is treated at a healthcare organization not affiliated with the TriNetX network and subsequently experiences a fatal outcome outside this network. However, this represents only a minor issue, as currently, 94% of healthcare organizations within the TriNetX network are also linked to the U.S. death registries. This percentage is steadily increasing as more healthcare organizations continue to be linked with the registries. Nevertheless, outcomes such as myocardial infarction or PTSD could be missed if the patient is followed up outside of the TriNetX network. Post-propensity matching cohort sizes varied slightly across outcome analyses due to TriNetX automatically excluding patients with documented outcomes occurring outside the predefined time window. The numbers of dropouts/excluded patients are reflected in Table 2.

In addition, there may be some issues with granularity in identifying which event occurred first on a specific date. It has been estimated that 20% of patients who undergo RSI on the same day as an ED visit have the intubation take place in the OR or ICU rather than the ED. Despite this limitation, identification of RSI exposure within the TriNetX platform demonstrates good positive predictive value, high specificity, and reasonable sensitivity. Validity is further strengthened through the combined use of procedure codes for intubation or mechanical ventilation, Medication Administration Record-linked RxNorm paralytic definitions within one day before or after, and documentation of an induction agent administered during the same encounter, collectively enhancing confidence in exposure classification. Evaluation of PTSD in this retrospective study may be limited as symptoms of PTSD may not be apparent or asked about during the initial hospitalization.

While we could not identify causality based on the retrospective nature of the study, the data points to additional avenues for evaluation. There is a limitation in attempting blinded randomized controlled trials in the setting of RSI, particularly under emergent circumstances, which would prove difficult if not impossible, as differences in physiological effects and timing would reveal the medication in use without speaking to the dangers of administrations in cases of contraindication to succinylcholine. Additional retrospective studies could be performed on the outcomes of the two drugs based on specific lab values, additional medical comorbidities, hospital environments and settings, the use of different induction agents, and indications for intubation; however, a post-hoc analysis showed only minor differences in intubations for trauma between the succinylcholine (25.7%) vs rocuronium (28.5%) groups.

We pulled data collected from multiple centers and settings but did not distinguish between them and may not account for all circumstances. Further, there are differences in pharmacokinetics, pharmacodynamics, side effects and adverse events for each medication, guiding clinician choice, and covariates not evaluated in the study. This study, based on CPT and *ICD-10-CM* codes rather than full chart review may represent a limitation; however, this is mitigated by the magnitude and multicenter design, which is six times larger than any previous study. There have been several changes in practice over the past 20 years including increasing use of video laryngoscopy, increasing use of non-invasive ventilation to avoid intubation, greater preoxygenation, and improved care for many disease processes that may influence the need for intubation.^17^,^18^ While this data was not well captured within this database, a post-hoc analysis was completed comparing succinylcholine to rocuronium in RSI patients from 2018–2025 showing similar results to our primary analysis.

Additionally, in most EDs, sugammadex as a reversal agent for rocuronium is not available due to costs and lack of established protocols, while it is readily available in most ORs.^24^–^27^ In 2024, sugammadex was the third highest hospital-based pharmaceutical expense.^28^ This may limit the generalizability of these findings to settings such as ORs where reversal agents are readily available. It should be noted that it takes approximately 2–3 minutes for sugammadex to reverse the paralysis from rocuronium. The reversal with sugammadex may be faster with higher dose administration or shorter time since the last dose of rocuronium.^26^

## CONCLUSION

Prior studies have attempted to evaluate outcomes between paralytic agents for rapid sequence intubation, but to our knowledge, none of them have established a significant difference in mortality between succinylcholine and rocuronium. This study, which is more than six times larger than previous studies, demonstrates an association with decreased mortality when succinylcholine is used over rocuronium. This study highlights the importance of evaluating real-world outcomes using large databases. The explanation for this association is probably linked to the shorter duration of succinylcholine’s effects and the challenges of maintaining oxygenation in a patient with prolonged paralysis, especially without a definitive airway. This study opens the door for further investigations into paralytic administration and suggests that succinylcholine might be safer in the absence of contraindications in the emergency department.

## Supplementary Information



## Figures and Tables

**Table 1:** Cohort characteristics, including demographics and pre-existing conditions associated with mortality, in a study comparing clinical outcomes of the use of succinylcholine vs rocuronium in emergency rapid sequence intubation (N = 30,189).

Cohort	Demographics	Before Propensity Matching			After Propensity Matching		
	
		Mean (SD)		P value	Mean (SD)		P value
1 Succ	Age at Index	54.5 (18.0)		< .001	56.0 (17.5)		.54
2 Roc		56.7 (17.3)			55.9 (17.5)		
	
		Patients	% Cohort	P value	Patients	% Cohort	P value

1	White	10,192	65.7%	.04	8,868	66.0%	.67
2		9,471	64.5%		8,835	65.7%	
1	American Indian or Alaska Native	47	0.3%	.06	47	0.3%	.92
2		64	0.4%		46	0.3%	
1	Female	6,068	39.1%	.67	5,253	39.1%	0.76
2		5,775	39.4%		5,277	39.3%	
1	Native Hawaiian or other Pacific Islander	57	0.4%	< .001	56	0.4%	.32
2		146	1.0%		46	0.3%	
1	Unknown ethnicity	1,068	6.9%	< .001	817	6.1%	.31
2		895	6.1%		857	6.4%	
1	Not Hispanic or Latino	13,045	84.1%	.21	11,299	84.1%	.51
2		12,262	83.6%		11,259	83.8%	
1	Hispanic or Latino	1,401	9.0%	< .001	1,326	9.9%	1
2		1,518	10.3%		1,326	9.9%	
1	Black	2,855	18.4%	.72	2,415	18.0%	.40
2		2,677	18.2%		2,468	18.4%	
1	Male	9,335	60.2%	.48	8,084	60.1%	.66
2		8,771	59.8%		8,049	59.9%	
1	Unknown race	1,119	7.2%	.04	941	7.0%	.76
2		968	6.6%		928	6.9%	
1	Asian	279	1.8%	< .001	279	2.1%	.24
2		406	2.8%		252	1.9%	
	Pre-existing Conditions						
1	Hypertensive diseases	6,269	40.4%	< .001	5,857	43.6%	.47
2		6,973	47.5%		5,926	44.1%	
1	Diabetes mellitus	3,132	20.2%	< .001	3,004	22.3%	.50
2		3,789	25.8%		3,050	22.7%	
1	Acute kidney failure and chronic kidney disease	3,022	19.5%	< .001	3,014	22.4%	.50
2		4,099	27.9%		3,060	22.8%	
1	Overweight and obesity	2,250	14.5%	< .001	2,162	16.1%	.23
2		2,763	18.8%		2,235	16.6%	
1	Heart failure	2,244	14.5%	< .001	2,172	16.2%	.61
2		2,797	19.1%		2,203	16.4%	
1	Cardiac arrest	198	1.3%	< .001	195	1.5%	.51
2		265	1.8%		208	1.5%	
1	Ischemic heart diseases	2,963	19.1%	< .001	2,806	20.9%	.39
2		3,511	23.9%		2,864	21.3%	
1	Malignant neoplasm of bronchus and lung	291	1.9%	.42	231	1.7%	.41
2		257	1.8%		249	1.9%	
1	Chronic lower respiratory diseases	3,913	25.2%	< .001	3,536	26.3%	.72
2		4,037	27.5%		3,562	26.5%	

*SD*, standard deviation; *Succ*, succinylcholine; *Roc*, rocuronium.

**Table 2:** Outcomes of death and myocardial infarction in a study comparing clinical outcomes of the use of succinylcholine vs rocuronium in emergency rapid sequence intubation, before and after propensity score matching (N = 30,189).

Outcome Before Propensity Matching				

	Succ (%) (n = 15,514)	Roc (%) (n = 14,675)	RR (95% CI)	P-value
Death	(n = 15,269) 4,472 (29.3%)	(n = 14,700) 5,006 (34.1%)	0.832 (0.832–0.889)	< .001
MI	(n = 14,700) 5,006 (34.1%)	(n = 13,413) 1,584 (11.8%)	0.862 (0.806–0.922)	< .001

Outcome After Propensity Matching				

	Succ (%)	Roc (%)	RR (CI)	P-value

Death	(n =13,213) 3,977 (30.1%)	(n = 13,266) 4,433 (33.4%)	0.901 (0.869–0.933)	< .001
MI	(n=12,377) 1,303 (10.5%)	(n=12,264) 1,454 (11.9%)	0.888 (0.828–0.953)	< .001

*Succ*, succinylcholine; *Roc*, rocuronium; *MI*, myocardial infarction; *RR*, risk ratio.
