# Supplementary material for: Outcomes of Succinylcholine and Rocuronium for Rapid Sequence Intubation in the Emergency Department
Source: West J Emerg Med. 2026 May 3;27(3):766–74. doi: 10.5811/westjem.50495 (PMC13246191; doi:10.5811/westjem.50495)
Supplement: Supplementary file 1 [file wjem-27-766-s001.docx]

**Supplementary Appendix**

Table of Contents

Greedy nearest neighbor matching (NNM)…………………………………..Page 2

TriNetX Information………………………………………………………….Page 3

Propensity Score Matching using Sci-kit Learning…………………………..Page 3

Supplementary Table 1…………………………...…………………………..Page 3

**Greedy nearest neighbor matching (NNM)**

The most common implementation of propensity matching is pair-matching, in which pairs of treated and control participants are formed. There are several common implementations of pair-matching. The most commonly used is greedy nearest neighbor matching (NNM), which we used, in which a treated participant is selected at random and then matched to the control participant whose propensity score is closest to that of the treated participant. The process is described as greedy because at each stage the control is selected who is closest to the currently considered treated participant, even if that untreated participant would serve better as a control for a subsequent treated participant. This process is then re-peated until a matched control participant has been selected for each treated participant. This process generally uses matching without replacement, so that once a control participant is matched to a treated participant, that control participant is no longer available for matching to a subsequent treated participant. A reﬁnement to NNM is NNM with a caliper restriction. Using this approach, a control participant is an acceptable match for a treated participant only if the diﬀerence in their propensity scores is less than a maximum amount (the caliper width or distance). For technical reasons, one typically matches on the logit of the propensity score and uses a caliper width that is deﬁned as a proportion of the (0.1-0.2) SD of the logit of the propensity score. A crucial step in any study that uses propensity score matching is to assess the degree to which matching on the propensity score resulted in the formation of a matched sample in which the distribution of baseline characteristics is similar between treated and control participants. This assessment is critical as it allows both the researcher and readers to assess whether matching on the estimated propensity score has removed systematic baseline diﬀerences between treatment. The use of the standardized diﬀerence, which is the diﬀerence in means in units of SD, is often used for assessing the similarity of matched treated and control participants. Some authors have suggested that a threshold of 0.10 (or 10%) be used to denote acceptable balance after matching^13^. Once acceptable balance has been achieved, analysts can unblind themselves to the outcome and compare outcomes between treated and control participants in the matched sample. The analyses conducted in the propensity score–matched sample can be similar to those that would be done in an RCT with a similar outcome.

**TriNetX Information**

Topaloglu U, Palchuk MB. Using a Federated Network of Real-World Data to Optimize Clinical Trials Operations. *JCO Clin Cancer Inform*. 2018;2:1-10. doi:10.1200/CCI.17.00067

**Propensity-Score Matching using Sci-kit learn**
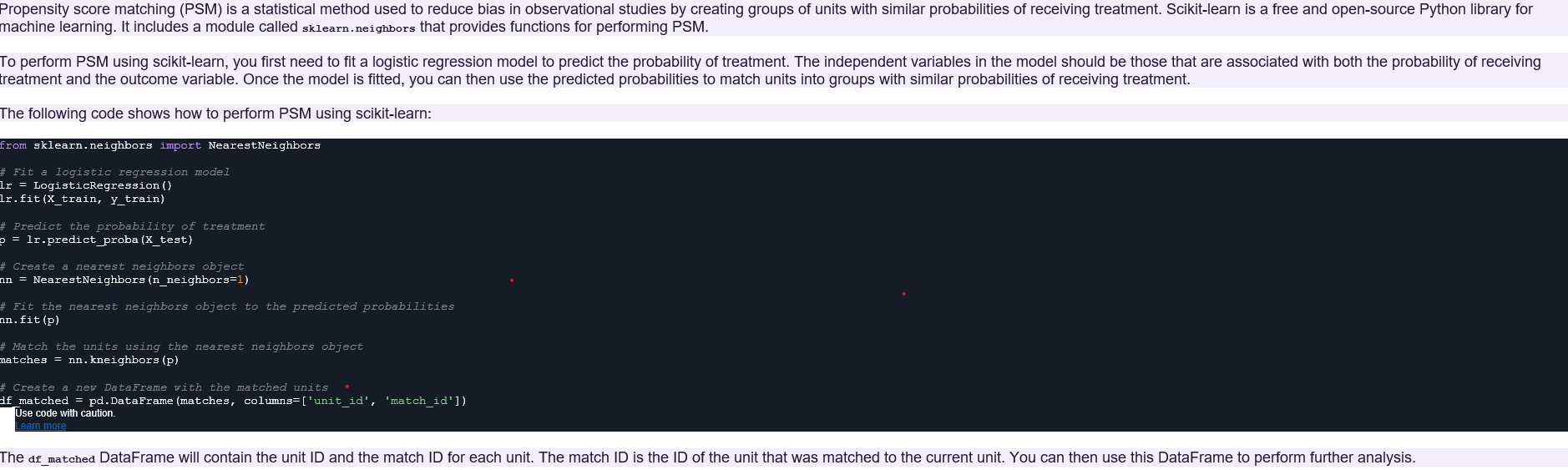


**Supplementary Table 1**

| **Day 0-60 Outcomes of Succinylcholine vs Rocuronium (2018-2025) in Patient given Etomidate or Ketamine** | | | | | | | | |
| --- | --- | --- | --- | --- | --- | --- | --- | --- |
|  | **Before Matching** | | | | **After Matching** | | | |
| **Outcomes** | **Succ** | **Roc** | **RR (95% CI)** | **P-Value** | **Succ** | **Roc** | **RR (95% CI)** | **P-Value** |
| Death | 4,780 (28.45%) | 8,512 (31.00%) | 0.92 (0.89,0.95) | <0.001 | 4,780 (28.46%) | 5,082 (30.25%) | 0.94 (0.91,0.97) | <0.001 |
| PTSD | 331 (2.07%) | 481 (1.84%) | 1.12 (0.98,1.29) | =0.101 | 331 (2.07%) | 331 (2.07%) | 1.00 (0.86,1.16) | =0.976 |
| MI | 1,797 (11.7%) | 3,255 (13.26%) | 0.88 (0.84,0.93) | <0.001 | 1,797 (11.70%) | 1,995 (13.06%) | 0.90 (0.84,0.95) | <0.001 |
| Succ = Succinylcholine, Roc = Rocuronium, RR = Risk Ratio, CI = Confidence Interval, PTSD = Post-Traumatic Stress Disorder, MI = Myocardial Infarction | | | | | | | | |
